# Comparison of the analgesic effect of intra-articular and extra-articular injection of morphine and ketamine compound in arthrotomy lower limb surgery under spinal anesthesia

**DOI:** 10.12669/pjms.305.4775

**Published:** 2014

**Authors:** Reza Akhondzade, Mohammad Reza Pipelzade, Mohammad Reza Gousheh, Naser Sarrafan, Kamran Mahmoodi

**Affiliations:** 1 Reza Akhondzade, Assistant Professor, Department of Anesthesiology, Ahvaz Jundishapur University of Medical Science, Pain Research Center, Ahvaz, Iran.; 2 Mohammad Reza Pipelzade, Associate Professor, Department of Anesthesiology, Ahvaz Jundishapur University of Medical Science, Pain Research Center, Ahvaz, Iran.; 3 Mohammad Reza Gousheh, Assistant Professor, Department of Anesthesiology, Ahvaz Jundishapur University of Medical Science, Pain Research Center, Ahvaz, Iran.; 4 Naser Sarrafan, Assistant Professor, Department of Orthopedics, Ahvaz Jundishapur University of Medical Science, Ahvaz, Iran; 5 Kamran Mahmoodi, Resident of Anesthesiology, Department of Anesthesiology, Ahvaz Jundishapur University of Medical Science, Pain Research Center, Ahvaz, Iran.

**Keywords:** Intraarticular, Extraarticular, Ketamine, Morphine, Post-operative pain

## Abstract

***Objective:*** One of the critical components in the postoperative care is pain. Given that little research has been done regarding the analgesic effects of intra-articular injection of ketamine, this study was aimed to compare the analgesic effect of intra-articular and extra-articular injection of morphine and ketamine compound in arthrotomy surgery under spinal anesthesia.

***Methods:*** A total of 50 patients were candidate for arthrotomy surgery, aged 18-60 years were divided randomly into two groups. At the end of surgery, the first group was treated with combination of intra-articular morphine and ketamine compound and the second group was treated with combination of extra-articular morphine and ketamine compound. The amount of postoperative pain was recorded in the hours of 2,4,6,12,24 respectively. Also 24 hours consumption of rescue analgesic was recorded.

***Results:*** The pain severity (VAS) in the hours of 2, 4, 6, 12 and 24 after surgery in the intra-articular injection group was significantly lower than the extra-articular injection group (P<0.05). Postoperative morphine consumption in intra-articular injection group (3.2±3.78) was significantly less than the extra-articular injection group (6.36±5.22) (p = 0.018).

***Conclusion:*** Postoperative pain severity of intra-articular injection of ketamine and morphine in knee surgery is less than extra-articular injection.

## INTRODUCTION

One of the critical components in the post-operative care of patients is acute post-operative pain control. After discovering the path of pain in the 19th century, significant improvements have been achieved in the field of pain management.^1^ The role of the anesthesiologist is key and important in this respect. At present, various methods are used for controlling the pain such as neuraxial blocks, opioid and non-steroidal anti-inflammatory drugs. Improper control of postoperative acute pain causes fatal complications of hemodynamic instability and increase the duration of hospitalization and cost of treatment.^2^^,^^3^ Due to the plethora of artrotomy surgery and severe pain in the postoperative stage, adequate pain control is necessary to accelerate the recovery movement of patient.^4^^-^^7^ Such methods include continuous epidural analgesia, femoral nerve block, Patient controlled analgesia (PCA) and intra-articular injection of local anesthetics or opioid drugs.^8^^-^^11^

Morphine and other opioids are used to control pain after surgery that have numerous side effects such as nausea, vomiting, respiratory depression, constipation, and loss of consciousness as well as the possibility of physical dependence and abuse, finding an alternative method has always been considered.^12^^-^^14^ In a study about intra articular injection of morphine performed in 2013 by Ozdemir et al. The authors concluded that intra articular morphine combined with levobupivacaine or bupivacaine decreases analgesic requirements and shortens the postoperative duration of analgesic use after knee arthroscopic surgery.^15^ In another study Arti et al. in 2013 showed the effectiveness of intra articular morphine in comparison to meperidine or methadone in pain relief after arthroscopic menisectomy.^16^ On of the other techniques for reducing pain was to reduce postoperative pain in knee surgery performed by Andersen et al. In their study ultrasound-guided saphenous nerve catheter was placed postoperatively in the adductor canal at midthigh level. They concluded that the combination of a continuous saphenous nerve block with single-dose local infiltration analgesia offered better pain relief on the day of surgery than local infiltration analgesia alone.^17^

Detection of N-Methyl-D-aspartate (NMDA) receptor and its role in reducing the pain has caused a new development in the use of ketamine medicine. A lot of reports have been published about the use of low dose ketamine as a NMDA receptor antagonist in relieving the pain and reducing the need for systemic opioids. This drug blocks NMDA receptors in the posterior horn postsynaptic membrane of the spinal cord and inhibits the pain transmission through the pain fibers to the central nervous system, resulting in reduced pain or no pain sensation. Recent research has shown that ketamine has a topical analgesic effect, as well.- Ketamine can be used in many routs such as intravenous, oral, intramuscular, intranasal, subcutaneous, or intra-anal or epidural and has rarely been used intra-articularly.

Given that little research has been done regarding the analgesic effect of intra-articular injection of ketamine and different answers are reported in this respect;^21^^,^^22^ and due to failure to achieve a clear answer about the effectiveness of this method, this study was conducted to compare the analgesic effect of intra-articular and extra-articular injection of morphine and ketamine in lower limb surgery under spinal anesthesia.

## METHODS

After obtaining approval from the Ethics Committee of Ahvaz Jundishapur University of Medical sciences (ETH-622), 50 patients candidate for arthrotomy lower limb surgery (knee) at ages 18-60 years, in ASA class 1 and 2 as inclusion criteria were randomly divided into two groups of 25 patients. The method of randomization was: Table of random numbers, computer-generated. Written informed consent was obtained from all patients. The first group was treated with a combination of morphine and ketamine compound by intra-articular injection and the second group was treated with the same compound extra-articularly. At the beginning of taking patients to the operating room, patients’ profile and the necessary information were collected using medical history records and as the exclusion criteria, all patients with a chronic history of narcotic or analgesic and patients who did not give consent to spinal anesthesia, were excluded from the study.

The amount of pain felt by the patient was measured separately using the visual analogue scale (VAS) index .At this scale, the number 10 represents the most severe levels of pain perception and zero indicates that the patient does not feel any pain.

Before spinal anesthesia appropriate monitoring (pulse oximetry, pressure gauge and ECG) were attached and 10 Kg / cc Ringer’s serum was infused. All patients underwent the spinal anesthesia by an anesthesiologist. Then the spinal anesthesia was performed by spinal needle No.25 "EXEL” brand in the L4-L5 orL3- L4 space. After insurance of detection of true space of subarachnoid and fluid output of cerebro-spinal fluid (CSF) 12.5mg hyperbaric bupivacaine was injected. Blood pressure in the first ten minutes, every three minutes, and then every five minutes was recorded and measured. If the systolic blood pressure was below 90 mm Hg or a decrease of 25% compared to the initial pressure, a 5-mg dose of ephedrine was injected. Patient’s sensory level was assessed through pin prick method, and surgery was permitted at the level of T10. Before surgery, the tourniquet pressure of 150 mm Hg above systolic pressure was closed, and at the end of the surgery and 10 minutes before opening the tourniquet of first group, morphine 5mg and ketamine 0.5mg/kg, dissolved in 20 ml of serum normal saline was injected intra-articularly, and in the second group morphine 5 mg and ketamine 0.5mg/kg, which dissolved in 20 ml of normal saline serum was injected extra-articularly. In intra-articular injection of knee, intra-synovial injection was administered and in the extra-articular injection, extra-synovial injection and inside the joint capsule (intra-capsular) was performed. Injections were administered by an orthopedic specialist. Sex, age, weight and the time of closed tourniquet of all patients were registered .The amount of postoperative pain were recorded at the hours of 2, 4, 6, 12, 24, by a person who did not know how to classify groups. If there was an analgesic request by the patient, 0.07mg/kg intravenous morphine was injected and the time of first analgesic request, and amount of post-operative opioid consumption was recorded as well.

The data were reported as mean ± SD. After checking the homogeneity of variances and normality of distribution of data, the independent T-test was used to compare postoperative pain and opioid consumption between the two groups in different hours (The power of study was 80%).

## RESULTS

Demographic characteristics of participants are shown in Table-I. The two groups were similar in terms of age, weight, and height. The mean first time of receiving morphine in post-operative intra-articular injection was 192±169.2 minutes and in the extra-articular group was 235.2±171 minutes and no statistical significant difference was observed between the two groups (P=0.45).The average pain severity (VAS) at 2, 4, 6, 12 and 24 hours after surgery in intra-articular injection group was significantly lower than the extra-articular injection (P <0.05), Fig.1.

## DISCUSSION

Pain as stress, induce psychological and physiological responses and the patient's response to pain is different, the consequences of pain have a direct effect on mortality and postoperative complications, recovery time and patient satisfaction from the health system.^23^ The varios form of opiates are used for pain control, it has advantages over the use of intra-articular for example, morphine when used over intra-articulary is more effective than it is used intravenously because of its low solubility in fat and Low joint blood flow, absorption will be less. In addition, there is less nausea and vomiting in intra-articular use.^24^ In terms of the average first time receiving morphine after the surgery, no statistical significant differences were observed (P= 0.45). The mean dose of postoperative morphine consumption in intra-articular group was significantly lower than extra-articular injection group (P = 0.018). The average pain severity (VAS) at 2, 4, 6, 12 and 24 hours after surgery in intra-articular injection group was significantly lower than extra-articular group (P<0.05). Solheim *et al. *in 2006 in Norway conducted a study, in which 60 patients were divided into two groups. Patients in the case group, received 5mg in 20ml of intra-articular morphine and control group received 20ml normal saline. In this study, no significant difference in the severity or pain relief between the two groups was observed within 48 hours after surgery. They concluded that intra-articular prescription of morphine 5mg has no significant clinical effect on the pain relief after knee arthroscopy surgery.^25^

Dal et al. in Turkey in 2004 examined the effects of intra-articular prescription of ketamine on analgesia after arthroscopic surgery. In this study, 60 patients were divided into four groups. The first group received ketamine 0.5mg/kg, the second group received neostigmine 0.5mg in 20ml, the third group received bupivacaine 20ml of 0.5%, and the fourth group received a placebo. They concluded that in the first three groups of postoperative pain, opioid consumption after surgery was significantly reduced. The study also showed that intra-articular prescription of ketamine provides extended and effective analgesia as neostigmine, but the impact is less than bupivacaine.^21^ Mntazeri *et al.* in a prospective randomized study in 2006 examined the effects of intra-articular injection of different doses of ketamine on pain severity after arthroscopic knee surgery. In terms of the amount of pain and time of asking for postoperative analgesia, no significant statistical difference existed between five groups. The results of this study showed that intra-articular injections of ketamine was not effective in reliving postoperative pain, opioid consumption and the time of ask for analgesic after diagnostic arthroscopy.^26^

**Table-I:** The data were reported based on mean ± standard deviation. There was no significant difference between the two groups (P<0.05).

***Height(cm)***	***Sex Ratio*** ***Female / Male***	***Weight(Kg)***	***Age (year)***	***Group***
167.29±5.41	2/23	74.19±6.14	31.48± 12.50	Intra-articular injection group
168.67±4.38	2/23	73.94 ±5.78	31± 12.52	Extra-articular injection group
71/0= p	1	0.43	0.89	Significance level (p.value)

**Figure-I:**
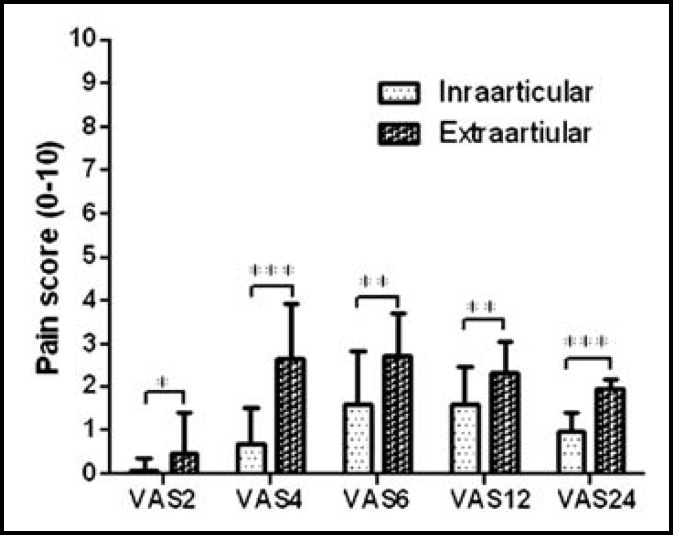
Pain intensity after surgery

The results of this study indicate the necessity for further studies on the use of intra-articular morphine and ketamine. The study examined the combined effect of both drugs in the intra-articular and extra- articular form, and similar to other studies of intra-articular ketamine, no side effects such as euphoria were found.

## CONCLUSION

Postoperative pain severity of intra-articular injection of ketamine and morphine in knee surgery is less than extra-articular injection.
